# 

**Published:** 1878-11

**Authors:** 


### THE BUFFALO MEDICAL AND SURGICAL JOURNAL.

VOL. XVIII. NOVEMBER, 1878. NO. 4.

